# Microevolutionary processes analysis in the Lithuanian genome

**DOI:** 10.1038/s41598-023-39249-5

**Published:** 2023-07-24

**Authors:** Laura Pranckėnienė, Alina Urnikytė, Vaidutis Kučinskas

**Affiliations:** Department of Human and Medical Genetics, Faculty of Medicine, Biomedical Science Institute, Vilnius University, Santariskiu Street 2, 08661 Vilnius, Lithuania

**Keywords:** Evolution, Genetics

## Abstract

Differences in the relative fitness of genomic variants are foundational, without these, neither natural selection nor adaption can exist. This research analyzed two microevolutionary forces, mutations, and positive selection, using whole genome sequencing data from Lithuanians across three generations: newborns (generation I), their parents (generation II), 60 years old Lithuanians, and the root ancestors (generation III). The main objective was to determine the frequency of mutations under selection in modern humans and how allele frequencies change across generations. Our results show that going through all the landscapes of the relative fitness on each chromosome, the general relative fitness background pattern remains the same in analysed generations. However, the tendency of relative fitness to decrease, in general, is noted. We hypothesize that the de novo genome variants or genome variants with a very low frequency that formed in the previous generation did not have time to be as affected by natural selection, thus, in the following generation, the force of natural selection acting on them is greater and their cumulative relative fitness also decreases. The strong natural selection pressure on the genetic regions that encode the *NEGR1* and *PTPN1/PTNP21* genes were also identified, highlighting the evolution of the Lithuanian population’s genome over generations, and possible genomic “deficiencies” for better adaptation.

## Introduction

The study subjects, Lithuanians, possess intriguing characteristics such as partial isolation, ancient genetic composition, and genetic differentiation within the European context^1^. After the last glaciation approximately 11,000 years ago, the initial settlers of Lithuania migrated to West Lithuania along the Baltic Sea^2,3^. These individuals originated from hunter-gatherer populations in Western Europe3. The formation of the first Baltic coastal culture in Lithuania occurred through the interaction between indigenous populations and Indo-Europeans during the late Neolithic period^4^. Archaeological, linguistic, and genetic evidence indicates an uncertain influence of the Finno-Ugric people on the Balts^1,2,4^. It has been proposed that around 6000–5000 years ago, during the middle Neolithic period, the Finno-Ugric people migrated to the eastern coast of the Baltic region. Until the late Middle Ages, the Eastern Baltic region remained one of the most isolated areas in Europe^5^. When the Roman Empire fell in the fifth century, the Eastern Baltic region was bypassed by the population movements of the Migration Period^1,6^. Later, during the First and Second World Wars, and the 1922–1945 and 1940–1952 emigrations, the exiles also had a significant impact on the population. From 1940 to 1952, Lithuania lost about 850 thousand people, i.e., almost one-third of the population7. By 1959, the Soviets brought about 214,000 residents of other nationalities to Lithuania. Lithuanian residents were moved from one place to another within the country in an organized manner^7^. Over the past 70 years, the size of the Lithuanian population significantly changed and shrank, and in 2022, it reached its former population size of 1960—only 2.8 million. From 1990 till now, the changes in population size have mostly been driven by economic emigration. Since the political and economic situation changes drastically over the past 50 years with the expansion and development of the food industry, depending on the people’s standard of living and geographical region, people’s diets have changed especially^8^. Life habits, such as physical activity, sleep patterns, and the level of stress experienced, have also changed. Medicine has been greatly improved, and the concept of personalized medicine has appeared^9^. The severity and rapidity of changes that drive evolution undoubtedly have affected and still affect the composition of the genome in a relatively short period of time. Thus, this context makes research on the microevolutionary process of the genome worthy of specific attention.

In recent years, there has been a significant focus on population genomic studies, investigating various evolutionary processes such as population structure, local adaptation, genetic admixture, and speciation with ever-increasing precision. These studies have unveiled a wide range of species responses to specific conditions. Concurrently, meta-analyses involving multiple species, often based on limited genome coverage data, have offered valuable insights into the ecological factors influencing genetic connectivity. These analyses have shed light on the impact of key life history traits on population structure. However, there remains a need for comprehensive integration of macro- and micro-evolutionary scales in comparative studies to fully unlock their potential^10^.

## Results

### Identification of positive selection signals

**Figure 1:**
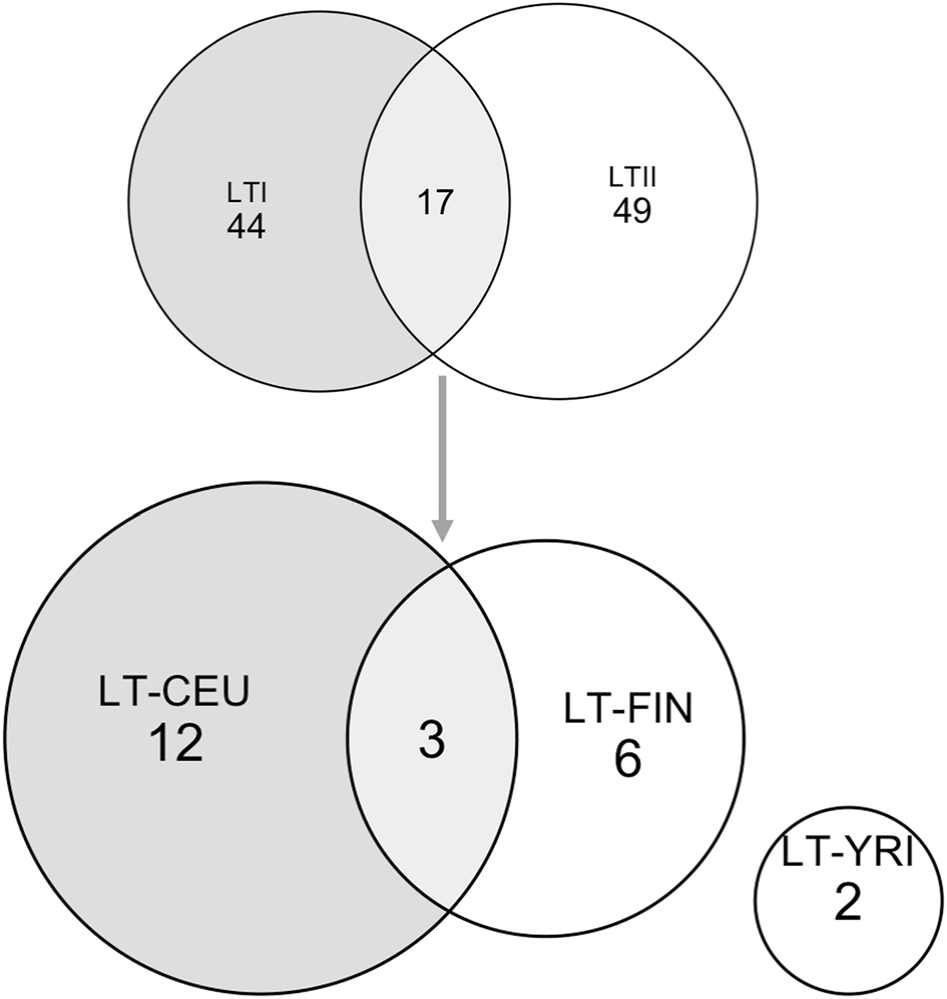
The Venn plot shows common candidate regions with signatures of recent selection passing from one generation (LTII) to another in the Lithuanian population (LTI); the number of shared signals of candidate regions for recent selection between Lithuanians (common regions of generation LTI and LTII, defined as LT) and the FIN, CEU, and YRI populations from the 1000 Genomes Project Phase3 dataset.

The genome-wide distribution signals for each comparison are summarized in Fig. S1. We detected 17 common candidate regions with signatures of recent selection passing from one generation (LTII) to another in the Lithuanian population (LTI) (Table 1, Fig. 1). Most recent signals were found when comparing Lithuanians (both generations common regions) to the CEU population (Fig. 1).

**Table 1:** Common candidate regions of selection detected with XP-EHH and *F*_*ST*_ for the Lithuanian population LTI and LTII.

	Genome coordinates	Genes	Population (SNPs^a^)
1	Chr1:21143733–21351053	*EIF4G3*	LTI/LTII*-CEU (5/9)
2	Chr1:143517337–143520326	*LOC102723769, RNVU1-1*	LTI/LTII-CEU (3/6)
3	Chr1:236836197–236926030	*HEATR1, ACTN2*	LTI/LTII-CEU (38/21)
4	Chr1:148754741–148759120	*NBPF25P*	LTI/LTII-FIN (4/19)
5	Chr1:117752105–117765366	*VTCN1, LINC01525*	LTI/LTII-YRI (13/26)
6	Chr2:98164532–98189967	*ANKRD36B*	LTI/LTII-CEU (55/55)
7	Chr3:75738871–75754988	*LINC00960, ZNF717*	LTI/LTII-FIN (16/20)
8	Chr3:171297866–171299145	*TNIK, PLD1*	LTI/LTII-CEU (14/14)
9	Chr3:110598818–110643608	*MIR4445, NECTIN3-AS1*	LTI/LTII-YRI (37/30)
10	Chr4:49220336–49,644,398	*CWH43*	LTI/LTII-FIN (40/39), LTI/LTII-CEU (27/34)
11	Chr6:32,541050–32554283	*HLA-DRB6, HLA-DRB1*	LTI/LTII-CEU (42/40), LTI/LTII-FIN (42/41)
12	Chr8:132247018–132280534	*ADCY8, EFR3A*	LTI/LTII-FIN (36/8)
13	Chr12:44279211–44301433	*TMEM117*	LTI/LTII-CEU (14/28)
14	Chr16:60012952–60015814	*LINC02141*	LTI/LTII-CEU (9/9)
15	Chr19:7042087–7061501	*MBD3L4, MBD3L2, MBD3L3, ZNF557*	LTI/LTII-CEU (5/3)
16	Chr20:48988616–48999918	*LINC01271, PTPN1*	LTI/LTII-CEU (14/9)
17	Chr22:20627265–20657642	*FAM230G, LOC107987389, FAM230J, FAM230G, LOC107987389*	LTI/LTII-CEU (7/8), LTI/LTII-FIN (7/3)

^a^Number of significant SNPs that were located at the extreme 0.1% of the empirical distribution for XP–EHH, and at least one SNP in the region had an *F*_*ST*_ p-value < 0.01, for LTI and LTII generations, separated by slash.

*LTI/II—first generation (newborns), and second generations (parents) of the Lithuanian population samples.

Biological pathways for genes near the targets of selection included genes that are involved in immune function (*HLA-DRB1*, *FBXL7*, and *PLD1*), metabolism (*PLD1*), cellular response to stimuli (*TNIK*), infectious disease (*ADCY8*), muscle contraction (*ACTN2*), and gene expression (*PTPN1*, *ZNF717*, and *ZNF557*). The terms identified using DAVID, of selected genes, are listed in Table S1. No significantly enriched terms were found in GO (FDR < 0.05) enrichment analysis.

A total of 28 strong candidate regions for older signatures of selection were identified in the two generations of the Lithuanian population when using the Tajima’s D statistic (Table 2). Those results were compared with Urnikyte et al. 2019^1^, who published the selection results, and analyzed the genotyping data of 424 60-year-old Lithuanian genome-wide high-density SNP genotype data, which could be considered as the third generation for comparison. In total, eight regions were found in all three generations (Table 2). Between these old selection signatures passing through the generations in Lithuanians were genes related to the efficient digestion of dietary fats, and in chromosome 10, comprising the *PNLIP* and *PNLIPRP3* genes that may probably result from local dietary selection pressures in the Lithuanian population. Other genes were related to olfactory receptors on chromosome 9, *OR1L1*, and *OR1L3*, the immune response on chromosome 11, *IL18BP*, vitamin D-binding (*GC*), and human skin color (*BNC2*).

**Table 2:** The candidate positively selected regions in three generations of the Lithuanian population, detected using Tajima’s D statistic.

**Genome Coordinates**	Windows	*p*-value	Genes	Generation
Chr1:86130328–86300327	7, 2	0.0009	*ZNHIT6*, *COL24A1*	LTI, LTII
Chr1:35713117–35873116	7, 4	0.0004	*SFPQ*, *ZMYM4*	LTI, LTII, LTIII*
Chr1:72043117–73453116	12, 11	0.0003	*NEGR1*, *LINC01360*	LTI, LTII
Chr3:112191045–112331044	10, 6	0.0002	*BTLA*, *ATG3*	LTI, LTII
Chr3:110510597–110931044	21, 31	0.0008	*MIR4445*, *NECTIN3-AS1*,* NECTIN3*	LTI, LTII
Chr3:128690597–129011044	21, 20	0.0004	*CFAP92*, *EFCC1*, *GP9*, *ISY1-RAB43*, *CNBP*	LTI, LTII, LTIII
Chr3:143470597–143651044	11, 10	0.0005	*SLC9A9*	LTI, LTII, LTIII
Chr4:72886863–73036862	11, 11	0.0004	*GC*, *NPFFR2*	LTI, LTII
Chr4:176226863–176396863	18, 15	0.0060	*ADAM29*, *GPM6A*	LTI, LTII, LTIII
Chr5:15302042–15502041	11, 12	0.0004	*LINC02149*, *FBXL7*	LTI, LTII
Chr5:171272042–171462041	8, 10	0.0030	*SMIM23, FBXW11*	LTI, LTII
Chr7:157314221–157434220	3, 5	0.0003	*LOC101927914, PTPRN2*	LTI, LTII
Chr8:42812883–43662883	22, 75	0.0060	*HOOK3, FNTA, POMK, HGSNAT, POTEA*	LTI, LTII
Chr9:16500870–16650869	9, 8	0.0009	*BNC2*	LTI, LTII
Chr9:125400470–125620469	13, 13	0.0030	*OR1L1, OR1L3, OR1L4, OR1L6*	LTI, LTII, LTIII
Chr10:118155879–118365878	12, 12	0.0004	*CCDC172, PNLIPRP3, PNLIP*	LTI, LTII, LTIII
Chr10:105935879–106225878	13, 20	0.003	*CFAP43*, *GSTO1*, *ITPRIP*, *CFAP58*	LTI, LTII
Chr11:71613588–71833587	7, 13	0.0008	*LOC100133315, RNF121, IL18BP, NUMA1*	LTI, LTII, LTIII
Chr12:60470077–60850076	29, 15	0.0020	*SLC16A7, TAFA2*	LTI, LTII
Chr12:80060077–80410076	26, 25	0.0030	*PAWR, PPP1R12A*	LTI, LTII
Chr13:48000628–48120627	3, 1	0.00021	*HTR2A, LINC00562*	LTI, LTII
Chr13:34140628–34510145	10, 10	0.0004	*STARD13, RFC3*	LTI, LTII, LTIII
Chr14:57690061–57860060	18, 3	0.0005	*EXOC5, AP5M1*	LTI, LTII
Chr14:61181447–61491446	29, 22	0.0005	*SIX4, MNAT1*	LTI, LTII
Chr15:64520639–65130638	52, 48	0.0040	*CSNK1G1, PCLAF, TRIP4, ZNF609, OAZ2, RBPMS2*	LTI, LTII
Chr18:18599491–18799490	12, 11	0.0002	*ROCK1*	LTI, LTII
Chr18:66591258–66871257	19, 16	0.0005	*CCDC102B, DOK6*	LTI, LTII

*LTI—first generation (newborns), LTII—second generations (parents), LTIII—60 years old Lithuanian population data taken from Urnikyte et al. 2019^1^.

Among the results based on Tajima’s D statistics, two significant (FDR < 0.05) Gene Ontology (GO) terms were identified: one BP term, and one molecular function (MF) term (Table S2). The enriched biological process was associated with DNA single-strand break repair, and the molecular function includes damaged DNA binding.

### The turnover of relative fitness for whole-genome variants

**Figure 2:**
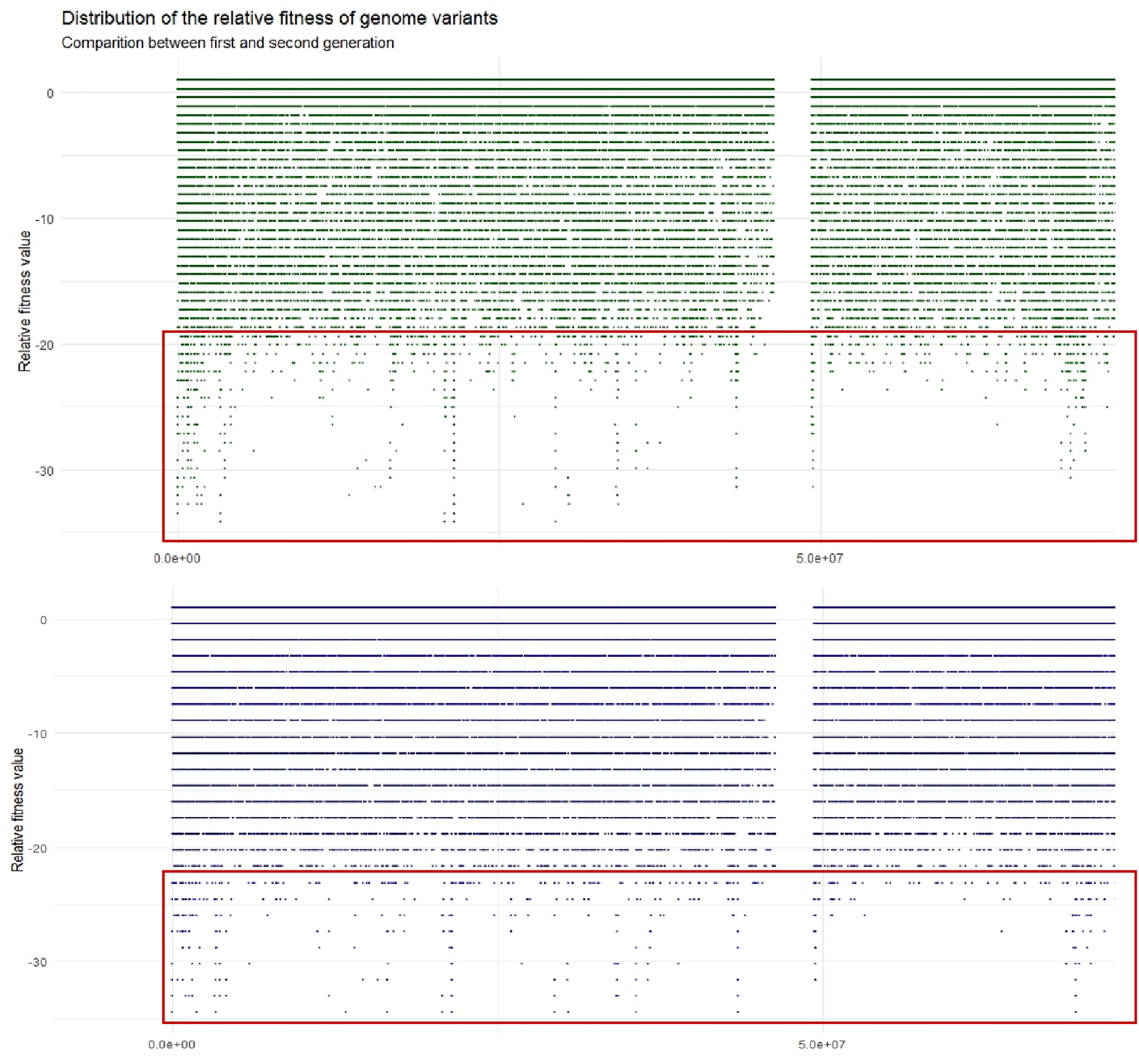
The part of the overlapping landscapes of relative fitness on chromosome 5. Green dots represent the values of relative fitness in the 2nd generation (LTII), and blue, in the 1st generation (LTI). The tendency for relative fitness to decrease is noted due to the higher density of the negative relative fitness values in the scale from − 20 and below.

Having each identified genomic variant frequency, we were able to evaluate the relative fitness values. Composing the values of relative fitness for each variant on a chromosome, the landscapes of relative fitness for each chromosome were formed (Fig. 2).

The analysis of the compared relative fitness landscapes between the second (LTII) and first generation (LTI) revealed 50 genomic regions (Table 3) where the relative fitness was significantly smaller or higher in the first generation than in the second one. Going through all the landscapes of relative fitness on each chromosome, the pattern of general relative fitness background remains the same in both generations. However, the tendency for relative fitness to decrease, in general, is noted (Fig. 2).

**Table 3:** Genomic regions were selected, with significantly altered values of relative fitness between 2 generations in the Lithuanian population.

**No**	Genome coordinates	Genes	Relative fitness
1	Chr1:70625000–72393850	*NEGR1*,* PTGER3*	Decreased
2	Chr2:130802518–133119329	*ARHGEF4*, *PLEKHB2*, *CCDC74A*, *GPR39*,* NCKAP5*	Decreased
3	Chr2:226875000–227318080	*COL4A4*, *COL4A3*,* RHBDD1*	Decreased
4	Chr3:72164948–72680412	*RYBP*	Decreased
5	Chr3:86340206–87371134	*POU1F1*	Increased
6	Chr5:16037735–16509433	*MARCHF11*, *RETREG1*,* ZNF622*	Decreased
7	Chr5:34183432–35708795	*TTC23L*, *RAI14*, *RAD1*, *BRIX1*, *DNAJC21*,* PRLR*	Decreased
8	Chr5:69230769–69476253	*CDK7*, *CCDC125*, *AK6*, *TAF9*,* MARVELD2*	Decreased
9	Chr5:78114009–78124999	*AP3B1*	Decreased
10	Chr5:96634615–96650000	*CAST*	Decreased
11	Chr5:137019230–137080000	*SPOCK1*	Decreased
12	Chr5:144811320–147169811	*PREIL2*, *GRXCR*, *SHR3F2*, *LARS1*, *RBM27*, *GPR151*, *PPP2R2B*,* TCERG1*	Decreased
13	Chr6:19940706–20108695	*RP3*, *RPL1*,* MBoAT1*	Decreased
14	Chr6:52650960–52690960	*TMEM14A*,* GSTA8P*	Decreased
15	Chr6: 120591518–125440090	*TBC1D32*, *GJA1*, *HSF2*, *PKIB*, *TRDN*, *NKAIN2*, *RNF217*, *TPD52L1*, *SMPDL3A*,* CLVS2*	Decreased
16	Chr7:19999999–20833333	*MACC1*	Decreased
17	Chr7:41249999–47916666	*GLI3*, *PPIA*, *ZMIZ2*, *MYL7*, *DBNL*, *URGCP*, *IGFBP3*, *CCDC201*,* C7ORF25*	Decreased
18	Chr7:124553571–124560000	*Noncoding sequence*	Decreased
19	Chr7:125400000–125450000	*Noncoding sequence*	Decreased
20	Chr7:161607140–166071420	*CRPPA*	Increased
21	Chr8:115591397–118279569	*TRPS1*, *EIF3H*, *SLC3OA8*, *MED30*, *EXT1*,* AARD*	Decreased
22	Chr9:17631578–17894736	*SH3GL2*	Decreased
23	Chr9:76339000–76339900	*PCSK5*	Decreased
24	Chr11:76694915–76699000	*GUCY2EP (pseudogene)*	Decreased
25	Chr11:85593200–85595000	*DLG2*	Decreased
26	Chr11:98728813–98729000	*Noncoding sequence*	Decreased
27	Chr11:106779600–106779700	*GUCY1A2*	Decreased
28	Chr12:19444440–19444900	*AEBP2*	Decreased
29	Chr12:46897900–46900000	*Noncoding sequence*	Decreased
30	Chr12:58500000–58550000	*Noncoding sequence*	Decreased
31	Chr12:80750000–80800000	*LIN7A*, *LINC01490 (RNA gene)*	Decreased
32	Chr13: 2525000–2530500	*Noncoding sequence*	Decreased
33	Chr13:76525423–82627118	*CLN5*, *ACOD1*, *KCTD12*, *SCEL*, *SLAIN1*, *EDNRB*, *OBI1*, *POU4F1*, *RBM26*, *NDFP2*,* SPRY2*	Decreased
34	Chr13:95593220–95783130	*DZIP1*,* DNAJC3*	Decreased
35	Chr14:30674157–31022727	*SCFD1*,* COCH*	Decreased
36	Chr14:79615384–79961900	*NRXN3*	Decreased
37	Chr14:88384615–94384615	*PTPN21*, *TTC8*, *FOXN3*, *DGLUCY*, *PPP4R3A*, *SLC2A4*, *NDUFB1*, *UNC79*, *PRIMA1*,* CCDC197*	Increased
38	Chr15:37666666–38444444	*TMCO5A*,* SPRED1*	Decreased
39	Chr15:64761904–66774193	*UBAP1L*, *PARP16*, *INTS14*, *MEGF11*, *SNAPC5*, *ZWILCH*,* LCTL*	Decreased
40	Chr16:51732673–53836633	*TOX3*, *CD9*, *RBL2*, *AKTIP*,* RPGRIP1L*	Decreased
41	Chr18:62446808–62447000	*Noncoding sequence*	Decreased
42	Chr19:1854838–1935483	*ABHD17A*, *ADAT3*,* SCAMP4*	Decreased
43	Chr19:47272728–48068181	*MEIS3*, *ZNF541*, *BICRA*, *SELENOW*, *TPRX1*,* TPRX2*	Decreased
44	Chr20:5508474–5762711	*GPCPD1*,* SHLD1*	Decreased
45	Chr20:6525423–6610169	*CAS20 (RNA gene)*	Decreased
46	Chr22:30092592–30099000	*HORMAD2*	Decreased
47	Chr22:32685185–32962296	*SYN3*,* TIMP3*	Decreased
48	Chr22:39256756–39864864	*ATF4*, *RPL3*, *SYNGR1, TAB1*, *MGAT3*, *MIEF1*, *CACNA1*,* ENTHD1*	Decreased
49	Chr22:48363636–48366000	*Noncoding sequence*	Decreased
50	Chr22:50000000–50878378	*IL17REL*, *TUBGCP6*, *NCAPH2*, *TYMP*, *ODF3B*, *CPT1B*,* RABL2B*	Decreased

The genomic regions where relative fitness differs from the background are distinguished by 134 protein-coding genes. The relative fitness is significantly decreased in genes that are involved in the numerous cellular processes that are initiated by extracellular stimuli that work through G protein-coupled receptors (*ARHGEF4*), signaling their intracellular transport (*MARCHF11*), which may be necessary for the long-term survival of nociceptive and autonomic ganglion neurons (*RETREG1*), the intrinsic apoptotic signaling pathway in response to oxidative stress (*ZNF622*), etc. Decreased relative fitness was detected in genes that are components of a heterotrimeric cell cycle checkpoint complex, known as the 9-1-1 complex, which is activated to stop cell cycle progression in response to DNA damage or incomplete DNA replication, also, in the *PRLR* gene, which may function to modulate the endocrine and autocrine effects of prolactin in normal tissue, and cancer or genes, the variants of which have been associated with *retinitis pigmentosa*.

Increased relative fitness was detected in genes that regulate the expression of several genes involved in pituitary development and hormone expression (*POU1F1*), the signaling pathway there coding protein PTPN21 regulates a variety of cellular processes including cell growth, differentiation, the mitotic cycle, and oncogenic transformation and insulin regulation (*SLC2A4*).

## Discussion

Our analyses demonstrate that distinct microevolutionary scenarios can generate very similar and realistic biodiversity patterns (e.g., the latitudinal diversity gradient). One of the biggest hits that we saw of selection was found in a ~ 131 kb region in chromosome 6, when comparing Lithuanian groups with CEU and FIN populations, which comprise the *HLA-DRB1* and *HLA-DRB6* genes, with the main function being to present pathogen-derived antigenic peptides to T lymphocytes. We identified three non-synonymous variants in the *HLA-DRB1* gene: rs9270302, NC_000006.11:g.32557479G > A, rs9270303, NC_000006.11:g.32557483 T > C, and rs707953, NC_000006.11:g.32557506 T > C. Lithuanians presented a high frequency (0.79) for the derived A allele at rs9270302, which is found at low frequencies in FIN (0.11), CEU (0.06), and YRI (0.29). The derived allele C at rs707953 also presents a high frequency (0.79) in Lithuanians and is found at intermediate frequencies in FIN (0.46), CEU (0.47), and YRI (0.50). The measured LD for these pairs of SNPs in plink^11^ showed complete LD between alleles. The frequencies of the variant rs9270302 were ~ 0.69 in CEU to 0.85 in Lithuanians. Fengxue Yu (2017) found that the variant rs9270303 was strongly associated with hepatitis B virus-associated hepatocellular carcinoma (HBV-HCC), however, its role still needs to be confirmed^12^. However, our findings provide fundamental data that need further study to confirm the roles of these variants. One of these hypotheses could be that those polymorphisms confer specific humoral immunity against common pathogens.

In some genes, we have identified non-synonymous variants. In the *COL24A1* gene: rs11161747 and NC_000001.10:g.86591837G > A may participate in regulating type I collagen fibrillogenesis at specific anatomical locations during fetal development^13^, in the *BTLA* gene: rs9288952, NC_000003.11:g.112185025G > A, with a function to inhibit lymphocytes during the immune response in the *PTPRN2*^14^ gene: rs1130495, NC_000007.13:g.157959911A > G, plays a role in vesicle-mediated secretory processes, and it is required for the accumulation of normal levels of insulin-containing vesicles and the prevention of their degradation, in the *OR1L4* gene, rs2215530, NC_000009.11:g.125486968G > A, and odorant receptor, and in the *PNLIP* gene: rs2915748, NC_000010.10:g.118313265T > C.

Another point of view of this study’s whole-genome analysis of microevolutionary processes was an analysis of the relative fitness turnover between two generations. Relative fitness shows how much fitness on a genotype has been compared to the maximum fitness, and so whether it will increase or decrease. Here, the relative fitness is a function not only of the individual, but also of all the generations in which they have been measured, and the relative fitness will change as the gene variant frequencies in the population change. Concerning the fitness of various sequence changes, not at the same speed as evolution occurs, the microevolution in the generations is an attempt to keep the most positive functional effect of each genomic variant in an ever-morphing landscape^15^. During this study, the aim was to find out how genomic and environmental elements determine the differences in relative fitness landscapes between generations, and in which direction the allele frequency changes from generation to generation in the Lithuanian genome. This study showed that going through all the landscapes of the relative fitness on each chromosome, the general relative fitness background pattern remains the same in both generations. However, the tendency of relative fitness to decrease, in general, is noted. We hypothesize that the de novo genome variants or genome variants with a very low frequency that formed in the previous generation did not have time to be as affected by natural selection, thus, in the following generation, the force of natural selection acting on them is greater and their cumulative relative fitness also decreases. Therefore, during the process of microevolution, the genome variants that are not adaptive enough are pushed out through time. Of course, we cannot claim that genomic variants will certainly be removed. On contrary, considering the effects of spatial variation^12,16^ in fitness and the fact that selection over many generations is a multiplicative process^17^, the genomic variant can become adaptive after all.

Surely, the comparison of relative fitness between the generations distinguished some specific genomic regions. Those genomic variants are necessary for the correct cellular signal transfer processes, DNA synthesis, and replication. In summary, the relative fitness decreased in the genes for which a mutation could significantly increase the risk of disrupting an important molecular process. A detailed description of gene functions is presented in Table S3. For example, the genomic variants in *ZNF622*, *PRLR* with decreased relative fitness show how important it is to protect an individual’s genome and to decrease variant rates in the genome: in the case of a *ZNF622* gene, if a mutation would be fixed in the genome, there would be a risk of having an imbalance between the reactive oxygen species and the antioxidant defense system. While it is known that oxidative stress is involved in most of the pathological states and diseases^18^, in the case of a *PRLR*, a fixed and potentially pathogenic genomic variant could disturb the modulation of the endocrine and autocrine effects of prolactin in normal tissue and cancer^19^, in the cases of *RP3* and *RP1*, it would disturb the structure or function of a protein that localizes to the outer segments of rod photoreceptors, and that is essential for their viability, mutations in this gene cause autosomal dominant *retinitis pigmentosa*. However, there was also an increase in the relative fitness detected in the genomic region, with the *TTC8* gene, whose mutations are also associated with *retinitis pigmentosa*. Therefore, this confirms what we have mentioned earlier, that in the general population, through microevolution, a cumulative relative fitness of genomic variants varies enough to maintain relative fitness equilibrium.

According to the data analysis results, regardless of the whole-genome analysis method—selection pressure analysis based on SNPs or relative fitness analysis on each identified genomic variant, a few genomic regions where *NEGR1* and *PTPN1/PTNP21* genes are placed, coincided. NEGR1 acts on the positive regulation of neuron projection development, and *PTPN1/PTNP21* codes PTPs that are known to be signaling molecules that regulate a variety of cellular processes, including cell growth, differentiation, mitotic cycle, and oncogenic transformation. The strong pressure of natural selection on these regions highlights the development of the genome of the Lithuanian population over generations, and possible genomic “deficiencies” for better adaptability. Since the relative fitness in the overlapping regions is not unambiguous—in the genome region where the *NEGR1* gene was identified, the relative fitness decreased, and in the case of *PTNP21*, it increased, this led to the conclusion that due to the reproducibility and complementarity of the results, both of the analysis methods used in this study are suitable for monitoring microevolutionary processes.

There are some limitations to this study. Because of the hypothesis-driven nature of this study, the sample size is relatively small due to economical limitations. In addition, more generations need to be included, which is impossible due to the human species. Despite all limitations, we have identified the candidate regions for selection in different Lithuanian generations, and the adaptive alleles that need to be validated.

In summary, in this study, we have shown that current macroevolutionary models may fail to distinguish between different microevolutionary scenarios. Therefore, establishing causal relationships between ecological factors and macroevolutionary rates or patterns requires rigorous evaluations. Future studies that incorporate microevolutionary processes into the current modeling approaches are needed.

## Materials and methods

### Sampling and DNA sequencing

We applied the SNP data of 25 trios from Lithuania (25 newborns, 25 mothers, and 25 fathers) obtained by WGS. Inclusion criteria, DNA extraction, WGS data processing were described previously^20^. All participants and their LAR/ parents provided informed consent. All experiments were performed in accordance with the Declaration of Helsinki, and all research methods were carried out in accordance with appropriate regulations and guidelines.

### Positive selection analysis

To detect recent signals of positive selection, our original genome sequencing data were merged with the data downloaded from the 1000 Genomes Project Phase3 dataset (gs://genomics-public-data/1000-genomes-phase-3, access in 2022)^21^. Data merging was performed with bcftools^22^ merge tool. SNP with > 20% missing data (max-missing) and SNPs with minor allele frequency (MAF) < 0.01 (minor allele frequency) were excluded. After merging we were left with 1,443,372 common SNPs. Haplotypes for the analysis were constructed with SHAPEIT2^23^. The signatures of recent or ongoing positive selection were investigated using the locus fixation index (*F*_*ST*_)^24^ and the cross-population extended haplotype homozygosity (XP-EHH)^25^. Both statistics were computed between the Lithuanian samples (generation I (LTI), newborns, and generation II (LTII), parents), and reference populations: related individuals, 99 Utah residents with Northern and Western European ancestries (CEU), 99 Finnish from Finland (FIN), and distant: 108 Yoruba from Ibadan (YRI)). The data of the generation III, 60 years old, Lithuanians were obtained from Urnikyte et al. 2019^1^. XP-EHH was run using selscan v1.2.0a^26^, and *F*_*ST*_ values were calculated with vcftools v.0.1.13^27^. For each comparison, an XP-EHH per SNP was obtained, and XP-EHH values of > 2 were considered as being indicative of selection. The SNPs located in the top 0.1% of the XP-EHH empirical distribution were considered as being significant ones. Significant regions were formed by combining significant SNPs that were less than 200 kb apart. We were interested only in those signals detected in the Lithuanian population samples. In each comparison, we considered as the top candidates for recent selection those genomic regions presenting at least two SNPs over the top 0.1% XP-EHH empirical values, and a minimum of one SNP with an *F*_*ST*_ rank score *p*-value of < 0.01.

The older signals of selection were inferred through Tajima’s D statistic^28^, and a calculation with the PopGenome^29^ package implemented in R v. 4.3.0 considering 100 kb sliding-window size and moving step of 10 kb^29^. Negative Tajima´s D values were identified considering the ranc of the score in the genomic distribution. For further analysis values with empirical p-value < 0.01 were used. P-values of all statistics were calculated using the rank of a score in the genomic distribution as described in Pybus M. et al. 2014^30^. The regions under selection were annotated with ANNOVAR^31^ using GRCh37 (hg19), dbSNP151^32^, RefSeqGene, and CADD (Combined Annotation Dependent Depletion), version 1.347^33^. The enrichment of biological processes in selected genes was tested using DAVID (Database for Annotation, Visualization, and Integrated Discovery)^34^ and Reactome v.3.7^35^. Linkage disequilibrium between SNPs were measured using plink v.1.07 the command –ld. Manhattan plots and a venn diagram were created with R v. 4.3.0.

### Structure of the relative fitness analysis

For relative fitness analysis, three groups of the general population without any additional health issues were analyzed. The third group consisted of the general European population (CEU, FIN, and YRI) for which data were derived from the 1000 Genomes Project Phase3 dataset^21^. This group was used as a reference generation no. 3 in this study (RIII). The second group was formed of adult individuals of Lithuanian origin (LTII)^20^. The first group of subjects are full-term healthy newborns from the general Lithuanian population, born in 2019–2020 (LTI).

Given the abundance of the identified variants for each group, all variants were grouped according to the genomic coordinates on the chromosomes. The calculation of relative fitness values was performed for the second and third generations in the study, comparing the frequency of each identified variant with the frequency of genomic variants in the “reference” first generation, regardless of its mechanism of formation. If the genomic variant that was identified in the second or third generation was not found in the “reference” first generation, then its frequency in the “reference” generation was considered to be the frequency of a single de novo mutation (1 × 10^−8^). The frequency of the genomic variant in the next generation is

$${{\mathrm{q}}_{1}}^{2}={{\mathrm{q}}_{0}}^{2}\frac{1-\mathrm{S}}{1-{\mathrm{Sq}}_{0}^{2}},$$

where ${{q}_{1}}^{2}$ is the frequency of the genomic variant in the second or third generation, ${{q}_{0}}^{2}$ is the genomic variant frequency in the “reference” first generation, and S is the strength of natural selection.

Additionally, from the genome sequencing data, we know the frequency of the genomic variants, and the strength of the natural selection that occurs through the generations is defined as follows:

$$S=\frac{{q}_{0}^{2}-{q}_{1}^{2}}{{q}_{0}^{2}-{q}_{0}{q}_{1}^{2}}.$$

With the calculated value of natural selection, the relative fitness was calculated as follows:

$${RF}_{w}=\frac{1-S}{1-{Sq}_{0}^{2}},$$

where RF_w_ is the relative fitness for each (*w*) identified genomic variant.

Visual Studio 2017 and C# language were used to write the calculation software, and a graphical presentation and analysis of the results was performed using the Rcmdr and *ggplo*t packages^36^.

### Ethics approval and consent to participate

This study was approved by the Vilnius Regional Research Ethics Committee, No. 2020/6-1243-724, date: 22-06-2022. All participants and their LAR/ parents provided informed consent.

## Supplementary Information

Supplementary Figures.

Supplementary Tables.

## Data Availability

The datasets analysed during the current study are available in the Figshare repository, doi: 10.6084/m9.figshare.22952774.
