# Supplementary material for: Microevolutionary processes analysis in the Lithuanian genome
Source: Sci Rep. 2023 Jul 24;13:11941. doi: 10.1038/s41598-023-39249-5 (PMC10366082; doi:10.1038/s41598-023-39249-5)
Supplement: Supplementary file 1 — Supplementary Figures. [file 41598_2023_39249_MOESM1_ESM.pdf]

Supplementary Material

Microevolutionary Processes Analysis in the Lithuanian Genome

Laura Pranckėnienė *; Alina Urnikytė* and Vaidutis Kučinskas

Department of Human and Medical Genetics; Biomedical Science Institute; Faculty of Medicine; Vilnius University; Santariskiu Street 2; LT-08661 Vilnius; Lithuania.

* Corresponding authors

Alina Urnikyte

E-mail: alina.urnikyte@mf.vu.lt

Tel: +370 69855292

Laura Pranckėnienė

E-mail: laura.pranckeniene@mf.vu.lt

Supplementary Figures

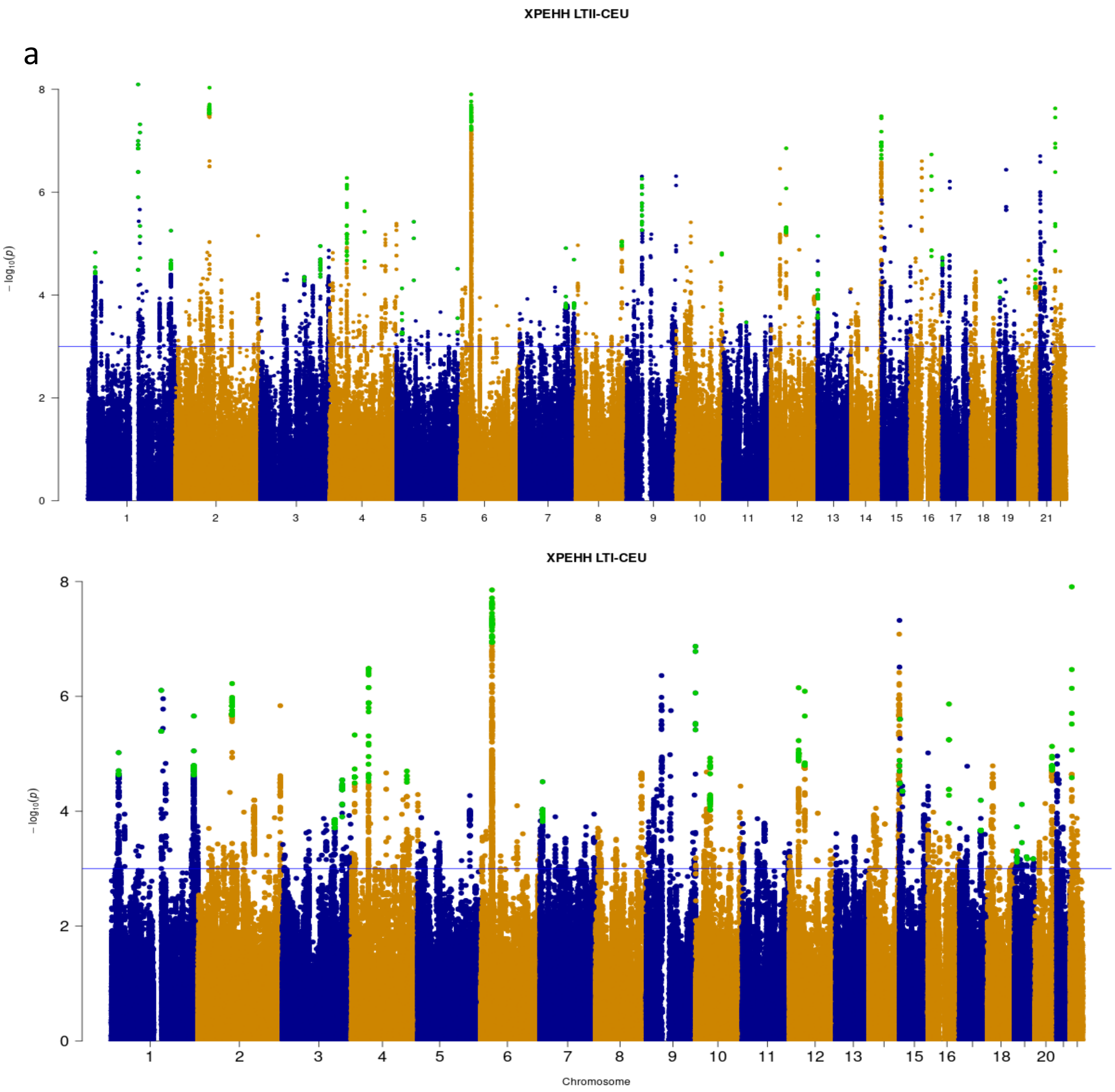

b

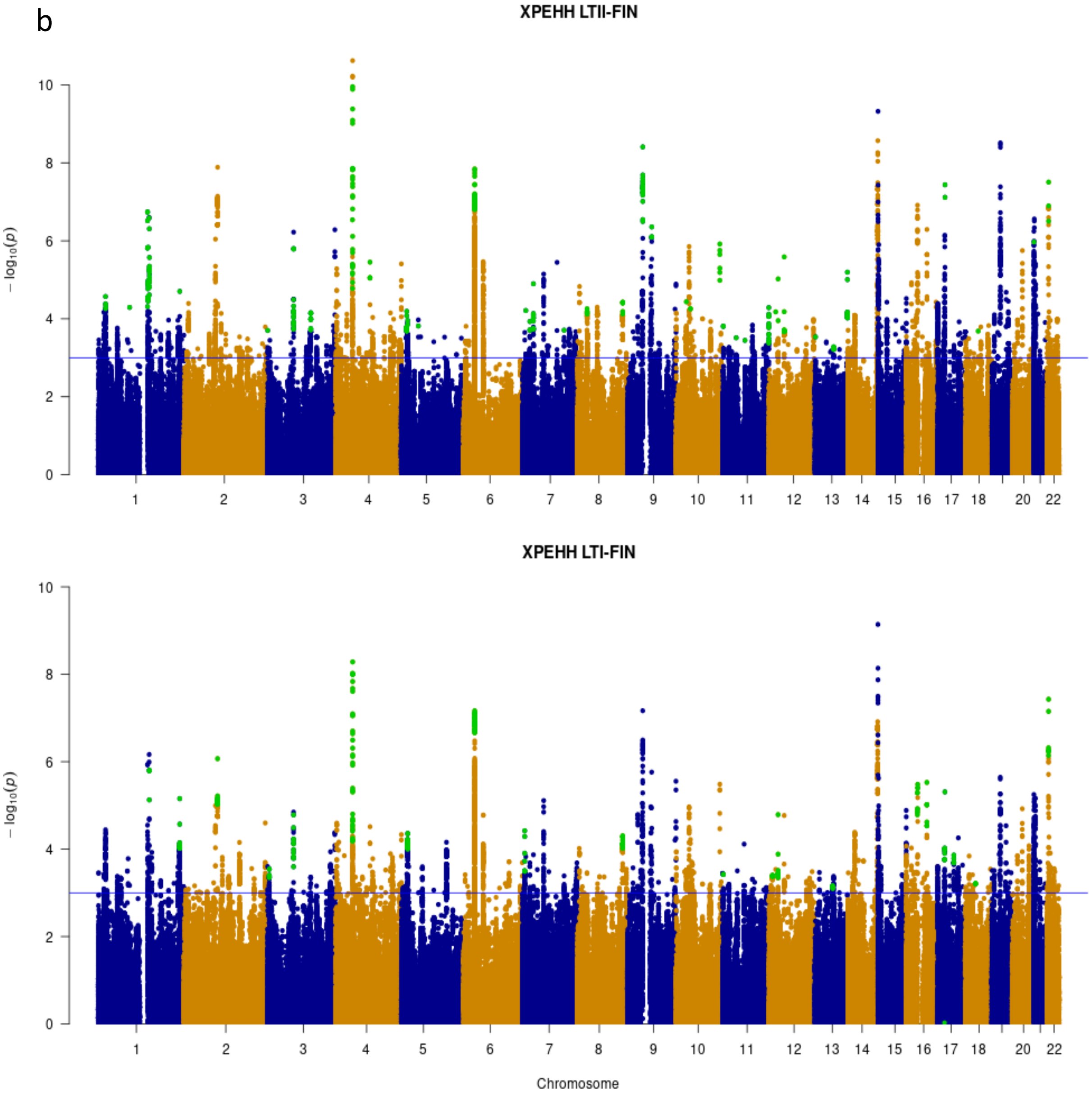

XPEHH LTII-FIN
$-\log_{10}(p)$
0
2
4
6
8
10
1
2
3
4
5
6
7
8
9
10
11
12
13
14
15
16
17
18
20
22
XPEHH LTI-FIN
$-\log_{10}(p)$
0
2
4
6
8
10
1
2
3
4
5
6
7
8
9
10
11
12
13
14
15
16
17
18
20
22
Chromosome

C

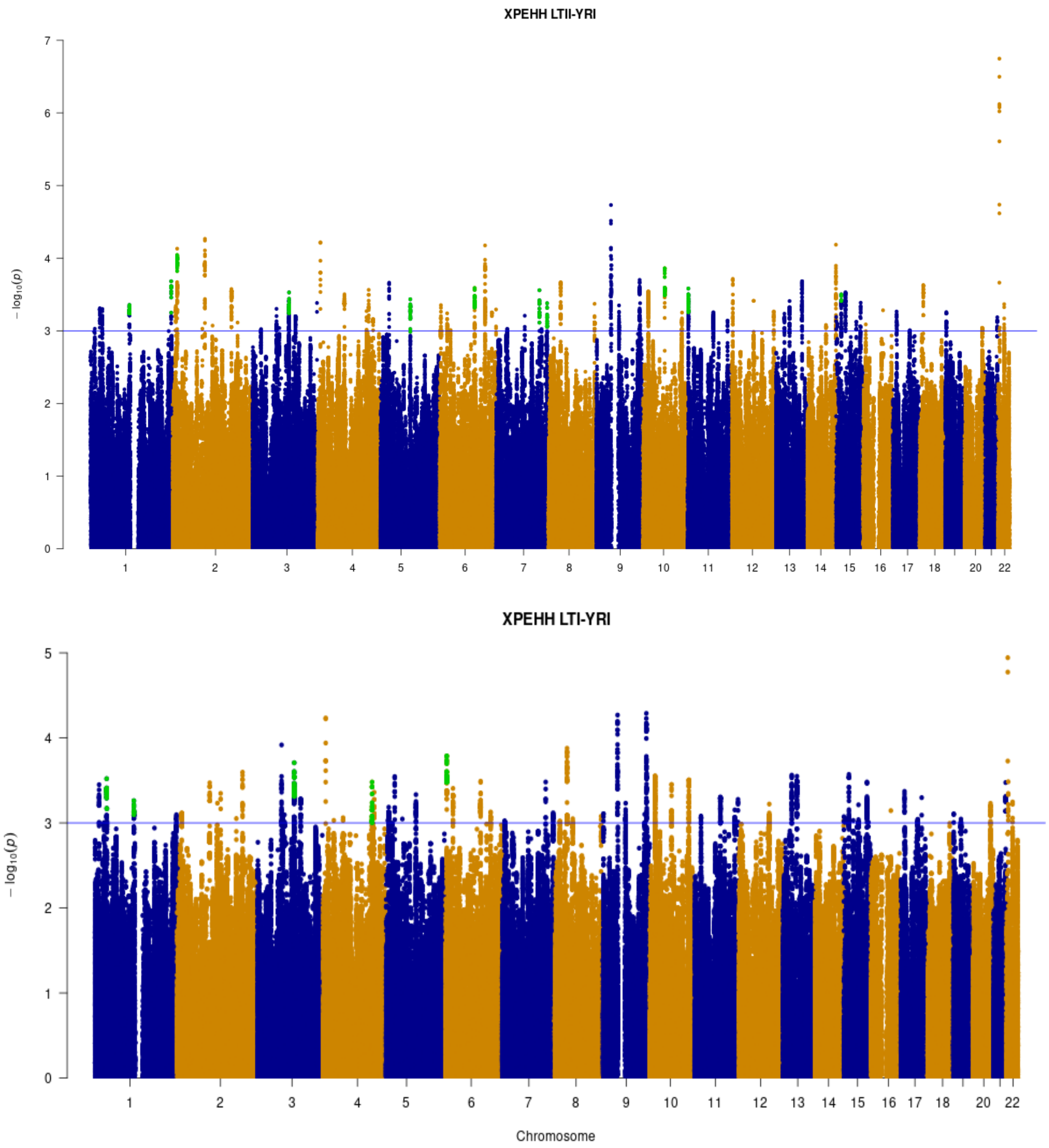

Figure S1. Manhattan plots of -log 10 transformed XP-EHH p-values across the autosomes. (a) XP-EHH in LTI/LTII-CEU, (b) XP-EHH in LTI/LTII-FIN, (c) XP-EHH in LTI/LTII-YRI. Green dots in each plot indicate those genomic regions presenting at least 2 SNPs over the top 0.1% XP-EHH empirical values and a minimum of 1 SNP with an FST rank score p-value < 0.01.

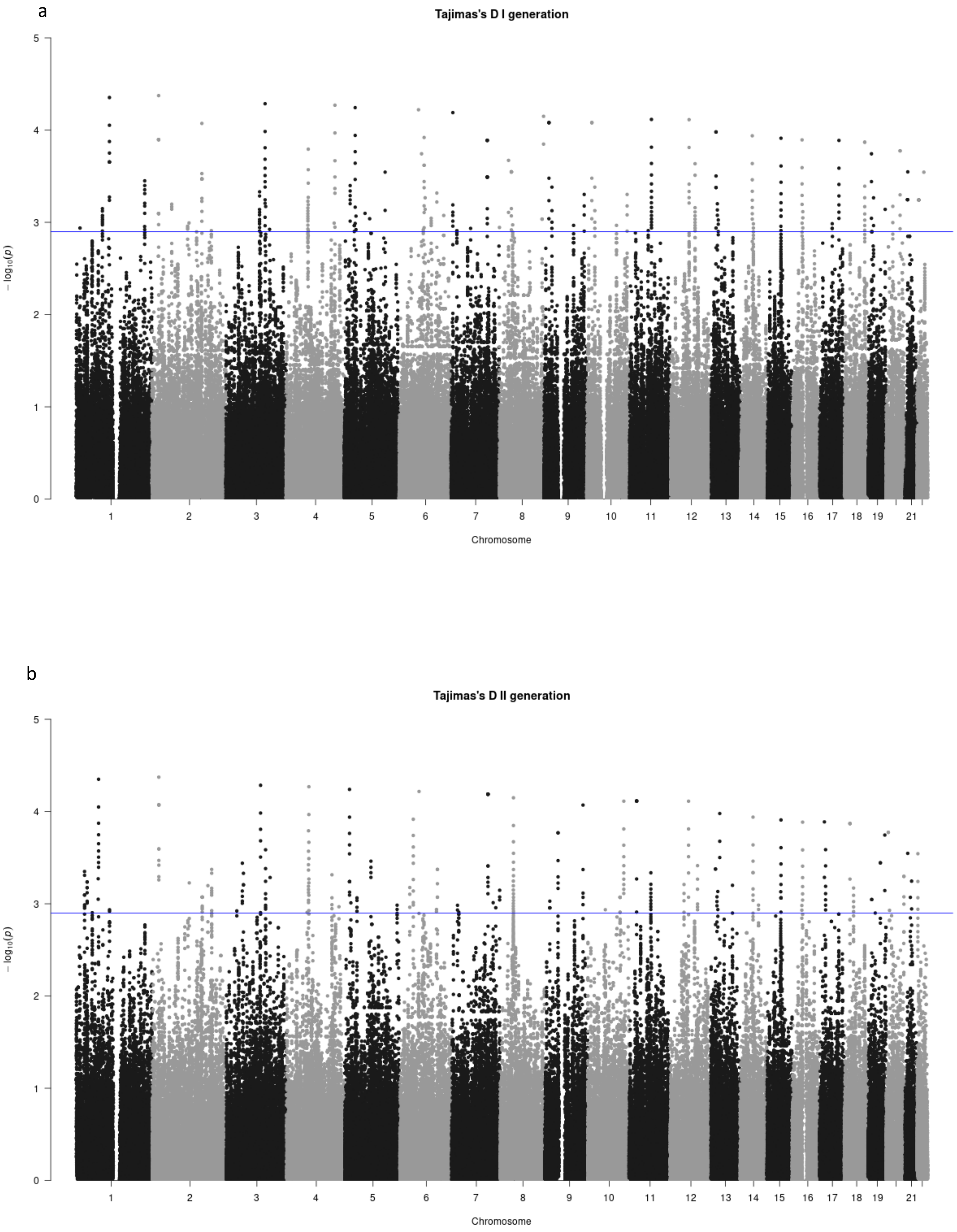

Figure S2. Manhattan plots of the log transformed Tajima's D p-values (a) In Lithuanians generation I, (b) In In Lithuanians generation II.
